# Targeted Microperimetry Grids for Focal Lesions in Intermediate AMD: PINNACLE Study Report 7

**DOI:** 10.1167/iovs.66.2.6

**Published:** 2025-02-04

**Authors:** Stefan Futterknecht, Philipp Anders, Julia Mai, Sophie Riedl, Ursula Hall, Chrysoula Gabrani, Kristina Pfau, Martin J. Menten, Daniel Rueckert, A. Toby Prevost, Hrvoje Bogunovic, Lars G. Fritsche, Ursula Schmidt-Erfurth, Sobha Sivaprasad, Andrew Lotery, Hendrik P. N. Scholl, Maximilian Pfau

**Affiliations:** 1 Institute of Molecular and Clinical Ophthalmology Basel, Basel, Switzerland; 2 Department of Ophthalmology, Universitätsspital Basel, Basel, Switzerland; 3 Vista Klinik, Binningen, Switzerland; 4 Laboratory for Ophthalmic Image Analysis, Medizinische Universität Wien, Wien, Austria; 5 BioMedIA, Department of Computing, Imperial College London, London, United Kingdom; 6 Institute for AI and Informatics in Medicine, School of Computation, Information and Technology, School of Medicine and Health, Technical University Munich, München, Germany; 7 Nightingale-Saunders Clinical Trials and Epidemiology Unit, King’s College London, London, United Kingdom; 8 Institute of Artificial Intelligence, Center for Medical Data Science, Medical University of Vienna, Wien, Austria; 9 Department of Biostatistics, University of Michigan School of Public Health, Ann Arbor, Michigan, United States; 10 National Institute of Health and Care Research Moorfields Biomedical Research Centre Moorfields Eye Hospital NHS Foundation Trust, London, United Kingdom; 11 Institute of Ophthalmology, University College London, London, United Kingdom; 12 University of Southampton Faculty of Medicine, Southampton, United Kingdom; 13 Current affiliation: Department of Clinical Pharmacology, Medizinische Universität Wien, Wien, Austria

**Keywords:** age-related macular degeneration (AMD), focal lesions, microperimetry, structure-function correlation, targeted microperimetry grids

## Abstract

**Purpose:**

The purpose of this study was to evaluate the feasibility and utility of optical coherence tomography (OCT)-based, targeted microperimetry grids in assessing focal lesions in intermediate age-related macular degeneration (iAMD).

**Methods:**

The multicenter, prospective PINNACLE study enrolled 395 patients with iAMD aged 55 to 90 years across 12 international sites. Participants underwent imaging, including OCT and microperimetry, every 4 to 12 months over 3 years. Deep learning algorithms detected focal lesions and changes in OCT images, including drusen regression, EZ/IZ loss with hypertransmission, and subretinal fluid, guiding 5-point microperimetry targeted to lesion locations. Data were analyzed using linear mixed models to estimate differences between retinal sensitivity measured by the 5-point focal grids and sensitivity interpolated from the 24-point standard grids.

**Results:**

The final analysis included 93 eyes from 83 patients, assessing 605 of the 5-point targeted grids and standard grids across 235 focal lesions. The Pearson correlation between focally measured sensitivity and interpolated sensitivity was 0.76. However, interpolation from the standard grid could be erroneous, especially in central regions of lesions characterized by EZ/IZ loss with hypertransmission and subretinal fluid. Interpolation errors increased with distance to the nearest measurement point (slope = 2.20 dB per degree, 95% confidence interval [CI] = 1.52 to 2.87). A significant negative relationship was found between interpolation errors and retinal sensitivity, with the highest errors in areas of low sensitivity. Lesion size significantly impacted interpolation errors for EZ/IZ loss with hypertransmission (slope = −19.41 dB/mm², 95% CI = −29.63 to −9.18).

**Conclusions:**

Targeted grids improved the detection and understanding of how focal retinal changes affect visual function in patients with iAMD, supporting the development of therapeutic interventions.

Age-related macular degeneration (AMD) is one of the leading causes of blindness worldwide.^1^^–^^3^ Although effective treatments exist for neovascular AMD, options for intermediate AMD (iAMD) are limited, and there is currently no treatment for the underlying degenerative disease.^4^ Best-corrected visual acuity (BCVA), which primarily represents the function of the fovea, does not adequately capture the progression of AMD, especially in cases where paracentral scotomata significantly impair visual function.^5^^–^^7^

Microperimetry enables spatially resolved assessment of retinal sensitivity and has shown a correlation with changes in the retinal pigment epithelium (RPE) and photoreceptors observed in optical coherence tomography (OCT).^7^^–^^12^ However, standard microperimetry grids may miss focal retinal lesions, thereby failing to provide a comprehensive assessment of retinal function.^10^

Recent advancements have led to the development of patient-tailored and targeted microperimetry grids for geographic atrophy (GA) and incomplete RPE and outer retinal atrophy (iRORA), which have shown promise in capturing the functional impact of specific retinal changes.^13^^,^^14^ Patient-tailored grids refer to individually customized test locations based on lesion morphology, whereas targeted grids, as used in this study, apply a standardized grid design to patient-specific locations. Despite these advancements, the functional impact of focal retinal lesions or events of change in iAMD on retinal sensitivity remains largely unknown. Moreover, a multicenter feasibility study of targeted microperimetry is lacking to date.

This study aimed to enhance the assessment of focal lesions in individuals with iAMD by introducing OCT-based, targeted microperimetry grids. Using these customized grids, the study intended to improve the detection and understanding of how focal retinal lesions affect visual function in patients with iAMD.

## Methods

### Study Design

The multicenter, prospective, noninterventional PINNACLE study enrolled 429 patients with iAMD (552 eyes, including both uni- and bilateral cases) across 12 participating centers, including the University Hospital Southampton and Moorfields Eye Hospital in the United Kingdom, the University Hospital Basel in Switzerland, and the Vienna General Hospital in Austria.^8^^,^^15^ The primary objective is to characterize and predict the progression of AMD using machine learning and advanced statistical modeling. Patients undergo a battery of imaging modalities, including OCT, OCT angiography (OCT-A), fundus autofluorescence imaging, adaptive optics imaging, and microperimetry every 4 to 12 months over 3 years, with yearly assessments of visual acuity outcomes. Focal events of change automatically detected by OCT prompt a targeted follow-up schedule, involving patient visits for targeted 5-point focal microperimetry measurements at 2 weeks, 4 weeks, and every 4 months post-detection, aimed at investigating these focal events in detail.

This study received ethical approval from the East Midlands – Leicester Central Research Ethics Committee (UK; ref. 19/EM/0163), the Ethics Committee of the Medical University of Vienna (Austria), and EKNZ (Swiss independent ethics committee). All procedures complied with Good Clinical Practice (GCP) principles, in line with the Declaration of Helsinki. Informed consent was obtained from all participants after explaining the nature and possible consequences of the study.

### Patients

In the PINNACLE study, subjects aged 55 to 90 years old with iAMD were included. The inclusion criteria required participants to have iAMD in both eyes, characterized by large drusen greater than 125 µm and/or definite hyper- or hypopigmentary abnormalities associated with medium or large drusen. Alternatively, subjects with iAMD in one eye (study eye) and advanced AMD (GA or choroidal neovascularization secondary to AMD) in the other eye were also included. All participants needed to have media clarity and pupillary dilation sufficient for adequate imaging and functional tests.

For this study report, we included all the patients in the PINNACLE study who experienced at least one focal lesion or event of change and had a 24-point standard microperimetry grid and a 5-point focal microperimetry grid measured on the same day.

The exclusion criteria, as outlined in Sutton et al. 2023,^15^ included any co-existent ocular disease affecting visual function or retinal morphology, established glaucoma with visual field or retinal nerve fiber loss, cataracts sufficient to affect imaging, and myopia ≥ −6 diopters. Patients who had undergone recent major ocular surgery or were taking drugs known to cause retinal toxicity were also excluded. Specific exclusions for the study eye involved OCT evidence of GA, complete RPE and outer retinal atrophy (cRORA), or choroidal neovascularization.

### Automated Spectral Domain-OCT Focal Lesion Detection

The automated detection of focal lesions and events of change was performed by the Laboratory for Ophthalmic Image Analysis at the Medical University of Vienna using advanced deep learning algorithms, as described previously.^16^^–^^18^ This process involved comparing subsequent OCT scans to identify retinal lesions and events of change, including focal drusen regression (> 90% volume loss over 125 µm of any drusen with a minimum height of 100 µm), ellipsoid zone/interdigitation zone (EZ/IZ) loss with hypertransmission (> 50 µm diameter), and new onset intra- or subretinal fluid.

The coordinates of these detected lesions were then sent to the study centers, where targeted microperimetry grids were designed based on this information. These customized grids were subsequently measured to assess the impact of the detected focal lesions on retinal sensitivity.

### Microperimetry

Patients underwent standardized microperimetry testing using a MAIA microperimeter (CentreVue, Padova, Italy). The MAIA microperimeter features Goldmann III stimuli with a diameter of 0.43 degrees, a dynamic range of 0 to 36 decibel (dB), a maximum luminance of 318.3 cd/m², and a background luminance of approximately 1.3 cd/m². The 4-2 staircase strategy was used to obtain thresholds. The device also provides real-time imaging and eye-tracking to ensure secure correspondence between the light stimulus and the retinal location. Mesopic testing was performed under standardized dim room lighting.

**Figure 1.**
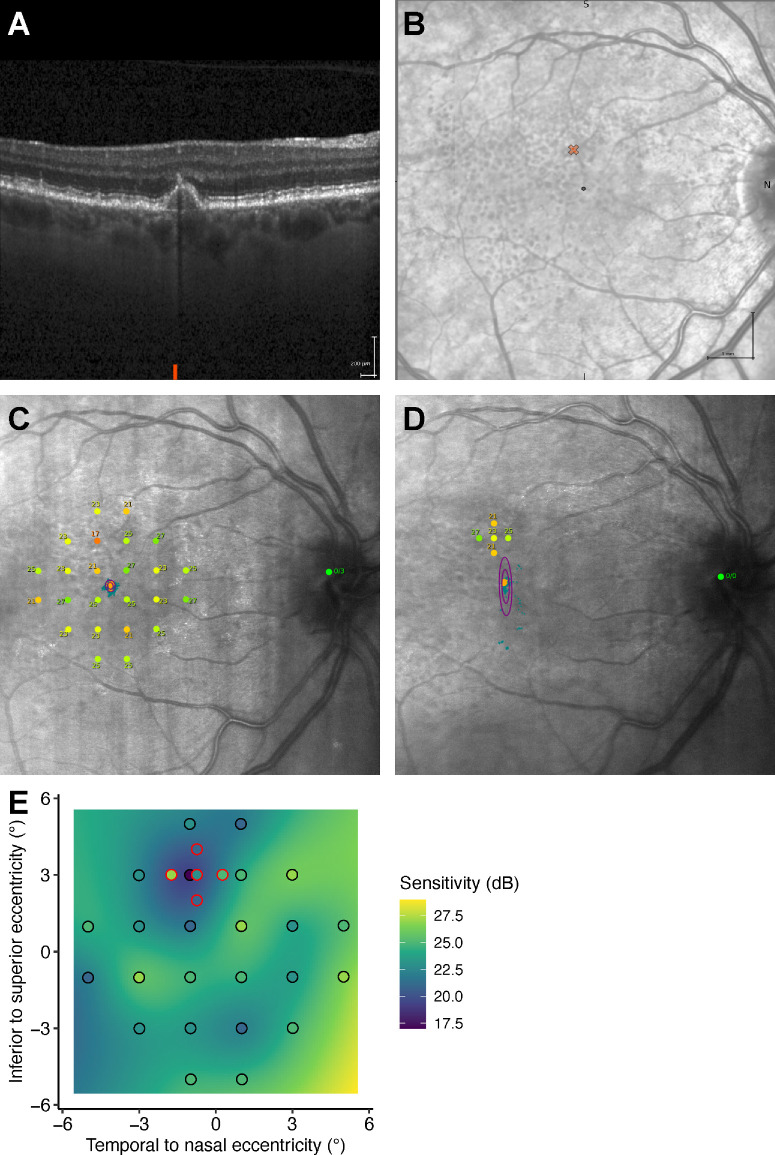
Methods. (**A**) An OCT scan highlighting an automatically detected focal lesion. (**B**) An infrared image generated during the OCT measurement, with the location of the focal lesion marked. (**C**) The infrared image taken during the 24-point microperimetry measurement overlaid with the 24-point microperimetry grid. (**D**) The infrared image taken during the 5-point microperimetry measurement, showing the 5-point focal grid positioned at the location of the focal lesion. (**E**) Background colors represent the predicted retinal sensitivity. *Points with black outlines* indicate retinal sensitivity measurements from the 24-point standard grid, whereas *points with red outlines* indicate measurements from the 5-point focal grid.

The grids used in the study included a 12-point training grid, presented at the beginning of every MAIA examination, and the 24-point PINNACLE standard grid, which was centered on the fovea and covered a central 10-degree diameter circle with 24 testing points located at −5 degrees, −3 degrees, −1 degree, 1 degree, 3 degrees, and 5 degrees on both the x-axis and the y-axis (Fig. 1C). This standard grid was performed yearly.

In addition to the standard grid, the 5-point PINNACLE focal grid was used for targeted follow-up when focal events of change were automatically detected by OCT (Figs. 1A, 1B). This involved patient visits for 5-point focal microperimetry measurements at 2 weeks after the focal events were detected and a second visit 4 weeks after the first targeted examination. Measurements were then repeated at all subsequent study visits. The 5-point focal grids were centered on the location of the focal lesions, with 1 central point and 4 points located superior, inferior, nasal, and temporal at a 1 degree distance from the central point (Fig. 1D). The grid design remained identical for all patients but was targeted to patient-specific locations based on the focal lesions identified by OCT. These grids were designed by the site investigator, imported as custom XML grids, and applied at all following study visits.

To validate the workflow, we measured the alignment of the OCT-defined target and the actual microperimetry examination in a random subset of 58 examinations. OCT infrared images and microperimetry infrared images were aligned using the Scale-Invariant Feature Transform (SIFT) algorithm in ImageJ (Supplementary Fig. S1). The distance between the location of the lesion in the OCT infrared image and the microperimetry infrared image was manually measured, revealing a mean offset of 0.75 degrees corresponding to 218.25 µm (Supplementary Fig. S2).

To obtain the local sensitivity at the incident focal event from the 24-point standard microperimetry grids, we applied thin-plate splines-based interpolation using the MGCV package in R (Fig. 1E). We optimized the hyperparameters k and λ, which define the “smoothness” of the interpolation, through a grid-search cross-validation approach to minimize the root mean square error (RMSE; Supplementary Fig. S3). The mean RMSE was found to be minimal with k set to 24 and λ set to −5, and these parameters were used for all subsequent analyses.

### Statistical Modeling and Analysis

Statistical modeling and analysis were conducted using R with the lme4, lmerTest, sjPlot, effects, and emmeans packages. A linear mixed model was used to estimate the differences (i.e. error as the dependent variable) between retinal sensitivity measured by the 5-point focal grids and the interpolated focal retinal sensitivity from the 24-point grids at corresponding locations within the same session. As potentially explanatory variables, we considered the lesion subtype, measured focal sensitivity, distance from the closest point in the 24-point grid, location in the 5-point grid, and lesion size, as well as 2-way interactions among these variables (Supplementary Table S1, Supplementary Equation S1). Random effects for patient-level clustering were included to account for inter-eye correlations, and repeated measurements were nested within subjects.

The location in the 5-point grid was included as an explanatory variable because the outer four points could extend beyond the lesion limits in small lesions (e.g. drusen).

For the linear mixed-model analysis, a stepwise backward selection was performed to identify the most relevant variables. The simplified models were compared to the initial model at each stage using an ANOVA with the Akaike information criterion (AIC) to ensure there was no significant loss of fit. All hypothesis tests were conducted at a 5% (0.05) significance level.

## Results

### Patient Demographics

In the final analysis, 93 eyes from 83 patients (73 patients were unilateral and 10 patients were bilateral) were included, each having both 5-point focal grids and 24-point standard grids measured during the same visit. The average age of the patients at baseline was (mean ± SD) 75.2 ± 6.9 years (Table 1).

**Table 1.** Patient Demographics

	Drusen Collapse	EZ/IZ Loss + Hypertransmission	Subretinal Fluid
Number of patients	32	71	7
Age, y			
Mean ± SD	74.3 ± 6.9	75.8 ± 7.0	70.1 ± 2.9
Gender, *n* (%)			
Female	24 (75.0)	46 (64.8)	7 (100.0)
Male	8 (25.0)	25 (35.2)	
Eye, *n* (%)			
Left	10 (31.2)	33 (42.3)	4 (57.1)
Right	22 (68.8)	45 (57.7)	3 (42.9)
Ethnic background, *n* (%)			
White	33 (100.0)	70 (98.6)	6 (85.7)
Asian		1 (1.4)	
Mixed			1 (14.3)
Smoker, *n* (%)			
Current smoker	1 (3.1)	3 (4.2)	2 (28.6)
Ex-smoker, > 1 mo	20 (62.5)	39 (54.9)	3 (42.9)
Never smoked	11 (34.4)	29 (40.8)	2 (28.6)
Lesions, *n*	56	171	8

*N*, number; SD, standard deviation.

For patients with drusen collapse (*n* = 32), the mean age was 74.3 ± 6.9 years. Among these patients, 75.0% were women, and 25.0% were men. Patients with EZ/IZ loss and hypertransmission (*n* = 71) had a mean age of 75.8 ± 7.0 years. This group included 64.8% women and 35.2% men.

Patients with subretinal fluid (*n* = 7) had a mean age of 70.1 ± 2.9 years. All patients with subretinal fluid were women.

### Focal Lesion Characteristics

A total of 605 of the 5-point focal measurements with 3025 measurement points were assessed across 235 individual focal lesions. The mean retinal sensitivity was 20.1 ± 4.6 dB, and the median lesion area was 16,657 µm² (interquartile range [IQR] = 4620 to 60,577). For drusen collapse, a total of 120 lesions were identified. The mean retinal sensitivity at these lesions was 22.2 ± 4.5 dB, with a median lesion area of 51,295 µm² (IQR = 19,019 to 184,818). In the case of EZ/IZ loss with hypertransmission, 464 lesions were observed. The mean retinal sensitivity at these lesions was 17.3 ± 7.6 dB, and the median lesion area was 5561 µm² (IQR = 2825 to 17,896]. Finally, for lesions with new onset subretinal fluid, 21 lesions were identified. The mean retinal sensitivity at these lesions was 15.8 ± 6.9 dB, and the median lesion area was 55,810 µm² (IQR = 24,564 to 563,178; Table 2). The spectral-domain OCT (SD-OCT) system used in this study featured a lateral resolution of 11.45 µm within each B-scan and an inter-B-scan spacing of 30.2 µm, enabling precise detection and analysis of small lesions.

**Table 2.** Focal Lesion Characteristics

Focal Lesion	Number of Measurement Points	Number of Focal Lesions	Sensitivity Mean ± SD, dB	Lesion Size Median [IQR], µm^2^
Drusen collapse	600	120	22.2 ± 4.5	51,295 [19,019–184,818]
EZ/IZ loss + hypertransmission	2320	464	17.3 ± 7.6	5,561 [2,825–17,896]
Subretinal fluid	105	21	15.8 ± 6.9	55,810 [24,564–563,178]

IQR, interquartile range; SD, standard deviation.

### Correlation of Interpolation-Derived and Measured Focal Retinal Sensitivity

The standard 24-point microperimetry grids were interpolated to match the locations of the 5-point focal grids. The Pearson correlation coefficient between the measured and interpolated focal sensitivity was 0.76, indicating a strong positive relationship (Supplementary Fig. S4).

### Interpolation Error Analysis

Across all lesions, the mean error between the interpolation-derived focal sensitivity in the 24-point standard grid and the measured focal sensitivity from the 5-point focal grid was 1.43 dB (95% confidence interval [CI] = 0.28 to 2.58).

Additionally, the fraction of interpolation errors of at least 7 dB was calculated, representing a retinal sensitivity difference beyond retest limits (considered clinically significant by the US Food and Drug Administration [FDA]^16^; Table 3).

**Table 3.** Interpolation Error Estimates

Focal Lesion	Location	Sensitivity, dB	Distance to 24-Points Grid, Degrees	Lesion Size, mm^2^	Interpolation Error, dB	SE	Lower 95% CI	Upper 95% CI	Fraction ≥ |7 dB|	Fraction ≤ −7 dB	Fraction ≥ 7 dB
Drusen collapse	Central	22.40	0.95	0.211	−0.26	0.56	−1.37	0.85	0.07	0.02	0.05
Drusen collapse	Peripheral	22.21	0.90	0.211	−0.13	0.56	−1.22	0.97	0.07	0.02	0.05
EZ/IZ loss + hypertransmission	Central	15.33	0.81	0.018	3.46	0.42	2.63	4.28	0.20	0.03	0.16
EZ/IZ loss + hypertransmission	Peripheral	17.75	0.90	0.018	1.54	0.41	0.73	2.35	0.14	0.05	0.08
Subretinal fluid	Central	15.10	0.84	0.731	2.30	1.29	−0.23	4.84	0.05	0.00	0.05
Subretinal fluid	Peripheral	15.93	0.80	0.731	1.61	1.28	−0.91	4.14	0.07	0.01	0.06

95% CI, 95% confidence interval; SE, standard error.

### Location in the 5-Point Focal Grid

**Figure 2.**
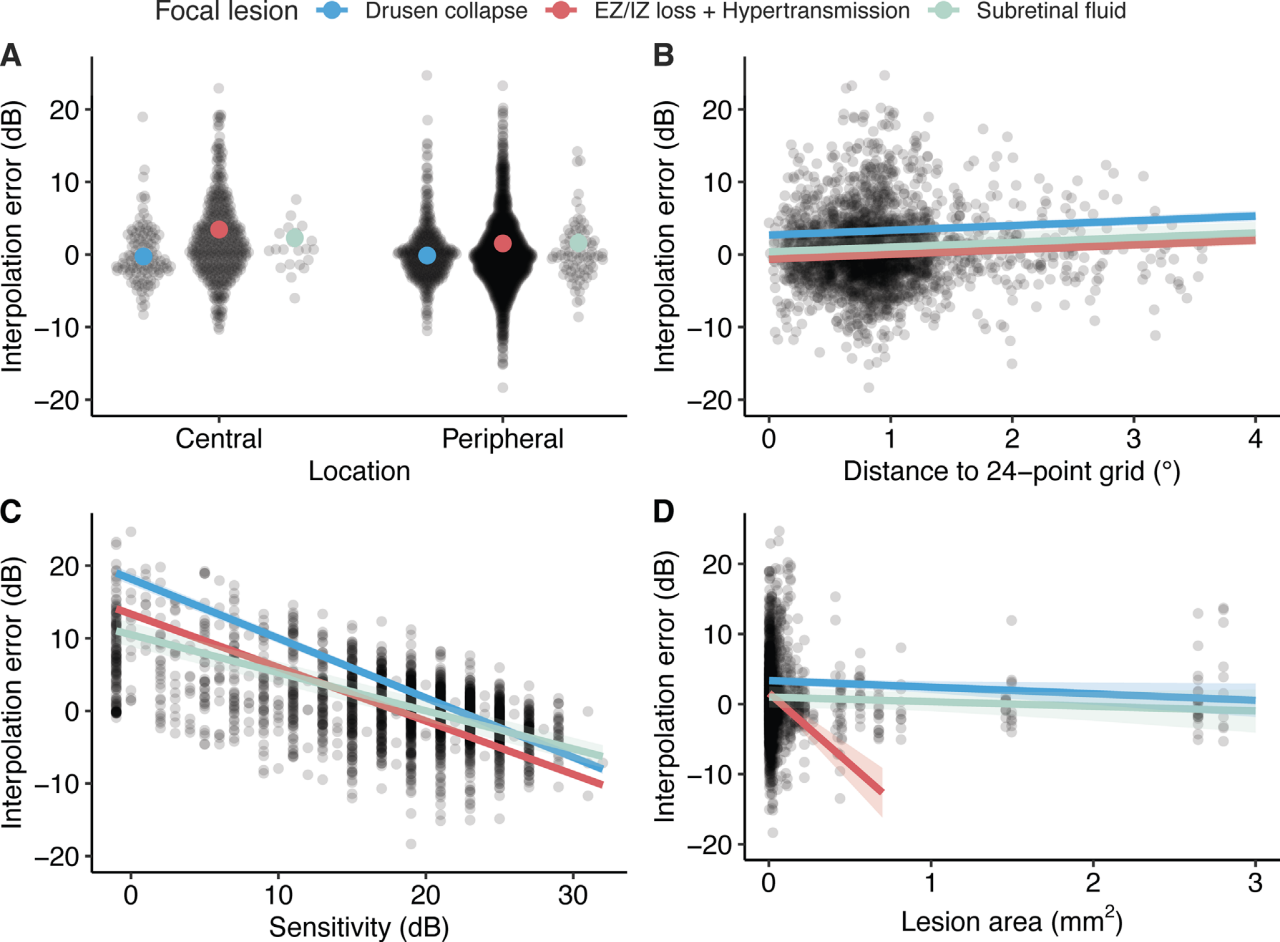
Results. (**A**) Interpolation error estimates categorized by the type of focal lesion and their locations within the 5-point focal microperimetry grid. “Central” refers to the central point in the 5-point grid (see Fig. 1D), whereas “peripheral” refers to the 4 peripheral points in the 5-point grid. (**B**) Interpolation error estimates based on the distance from the predicted point to the nearest point in the 24-point standard microperimetry grid. (**C**) Estimated slopes of interpolation error in relation to measured retinal sensitivity. (**D**) Estimated slopes of interpolation error in relation to the area of the lesion.

The interpolation error in relation to the location in the 5-point focal grid was analyzed (Fig. 2A). For drusen collapse in the central point of the grid, the mean interpolation error was −0.26 dB (95% CI = −1.37 to 0.85). The fraction of absolute errors greater than or equal to 7 dB was 7%. In the peripheral location, the mean interpolation error was −0.13 dB (95% CI = −1.22 to 0.97), with 7% of absolute errors greater than or equal to 7 dB.

In the case of EZ/IZ loss with hypertransmission in the central location of the grid, the mean interpolation error was 3.46 dB (95% CI = 2.63 to 4.28), indicating that interpolation-derived sensitivity markedly overestimates the measured focal sensitivity. The fraction of absolute errors greater than or equal to 7 dB was 20%. In the peripheral location, the mean interpolation error was 1.54 dB (95% CI = 0.73 to 2.35), with 14% of absolute errors greater than or equal to 7 dB.

For subretinal fluid lesions in the central location, the mean interpolation error was 2.30 dB (95% CI = −0.23 to 4.84). The fraction of absolute errors greater than or equal to 7 dB was 5%. In the peripheral location, the mean interpolation error was 1.61 dB (95% CI = −0.91 to 4.14), with 7% of absolute errors greater than or equal to 7 dB.

These results indicate the largest interpolation errors in the central regions of EZ/IZ loss with hypertransmission and subretinal fluid and low overall interpolation errors in case of focal drusen regression. The highest fraction of clinically relevant absolute errors was observed in the central regions of EZ/IZ loss with hypertransmission accounting for 20% of all interpolations.

### Distance to 24-Point Grids

The influence of the distance of the focal grid measurement points to the closest point in the 24-point standard grid was investigated. The mixed-model analysis revealed a significant relationship between the interpolation error and the distance to the closest measurement point of the standard 24-point grid (Fig. 2B). The analysis showed a positive slope of 2.20 dB per degree distance (95% CI = 1.52 to 2.87), indicating that the interpolation error increases (i.e. overestimation of retinal sensitivity) as the distance from the measurement points used for interpolation increases.

### Retinal Sensitivity

The impact of retinal sensitivity on interpolation error was examined. The results indicated a significant negative relationship between the interpolation error and retinal sensitivity (Fig. 2C). Specifically, the highest positive errors, indicating overestimation, were observed in lesions with very low retinal sensitivities, whereas the smallest negative interpolation errors, indicating underestimation, were found in lesions with very high retinal sensitivities. The slope for drusen collapse was −0.77 dB per dB of sensitivity (95% CI = −0.83 to −0.71). For lesions with EZ/IZ loss with hypertransmission, the slope was significantly flatter by 0.08 dB per dB of sensitivity (95% CI = 0.03 to 0.14). Similarly, the slope for subretinal fluid was 0.29 dB per dB of sensitivity (95% CI = 0.20 to 0.39) flatter. The interpolation error was the smallest in the retinal loci with a sensitivity of 19.06 dB.

### Lesion Size

The effect of the lesion size on the interpolation error was studied. The linear mixed-model analysis showed a significant negative correlation between the interpolation error and lesion size for lesions with EZ/IZ loss with hypertransmission, with a slope of −19.41 dB/mm² (95% CI = −29.63 to −9.18). In contrast, this relationship was not statistically significant for focal drusen regression (–0.93 dB/mm^2^, 95% CI = −2.60 to 0.74) or subretinal fluid (0.27 dB/mm^2^, 95% CI = −2.60 to 3.15; Fig. 2D).

## Discussion

Recent evidence suggests that complement inhibition may not only slow RPE atrophy secondary to AMD but could also target early forms of photoreceptor degeneration.^19^^,^^20^ This finding implies that the prevention of photoreceptor degeneration in iAMD might be achievable. However, the success of such interventions hinges on the availability of validated functional end points, which are essential for conducting clinical trials in iAMD. Our study provides robust evidence supporting the use of OCT-based, targeted microperimetry grids as a sensitive tool for detecting and characterizing focal retinal dysfunction in iAMD. By using grids that specifically target structural features identified via OCT, we were able to detect localized sensitivity losses in regions vulnerable to disease progression – areas that standard microperimetry grids often fail to assess adequately. This enhanced detection of early dysfunction is crucial, as subtle changes in retinal function can serve as early markers of disease progression, potentially guiding timely therapeutic interventions.

Our analysis revealed that the difference between the retinal sensitivity measured by the focal grids and the sensitivity interpolated from the standard grids varied significantly depending on the type and location of the focal lesion. The largest interpolation errors were observed in the central regions of lesions characterized by EZ/IZ loss with hypertransmission and subretinal fluid. The interpolation error in the case of EZ/IZ loss with hypertransmission corresponds approximately to the sensitivity loss associated with nascent GA.^14^^,^^21^^,^^22^ This is plausible, given that EZ/IZ loss with hypertransmission and nascent GA describe similar underlying lesions despite their different definitions.^23^ This also aligns with previous structure-function analyses highlighting the strong relationship between mesopic sensitivity and integrity of the outer retinal laminae in SD-OCT.^7^^,^^9^ Subretinal fluid exhibited a smaller effect on the interpolation error, which is likely linked to the established lesser effect of subretinal fluid on retinal sensitivity.^24^ Conversely, drusen regression showed low overall interpolation errors, indicating that isolated drusen regression has (at least initially) only a low impact on focal retinal sensitivity.

Importantly, lesion size and measured focal retinal sensitivity were predictors of the interpolation error for EZ/IZ loss with hypertransmission. This means that hill-of-vision interpolation overestimates retinal sensitivity the most for small lesions with deep scotomata. Within a standard 24-point microperimetry grid, where the measurement points are spaced 2 degrees apart and 0.43 degrees in diameter, a circular lesion of size 0.20 mm² (equivalent to a radius of 0.785 degrees or approximately 225 µm) could remain undetected.

In contrast, lesion size did not significantly impact the interpolation errors for focal drusen regression or subretinal fluid. These lesions were typically much larger than those with EZ/IZ loss with hypertransmission. Especially for drusen regression, our results indicate that the standard microperimetry grid sufficiently captures the functional impact of these lesion types.

Limitations in this study include the alignment of OCT and microperimetry measurements. Although the alignment was validated with a relatively small mean offset of 0.75 degrees, minor image alignment discrepancies could still impact the accuracy of retinal sensitivity measurements, especially in very small lesions. Additionally, recent findings using adaptive optics microperimetry (20 µm stimulus diameter) have shown that MAIA-based microperimetry may significantly underestimate local sensitivity loss due to its larger stimulus size and less precise tracking.^25^ Whereas the automated detection of focal lesions using deep learning algorithms is advanced, it may still produce false positive or negative results, affecting the reliability of lesion identification and subsequent analysis. Moreover, our use of focal grids was initiated after focal events were detected. Ideally, grid placement should be performed pre-emptively by using artificial intelligence (AI) approaches to forecast AMD progression.^26^ Finally, for drusen regression, the follow-up period may not have been long enough to differentiate drusen regression associated with RPE disruption from regression that leaves the retina apparently intact.

In conclusion, targeted microperimetry grids allow for a more nuanced assessment of retinal sensitivity in areas affected by focal lesions, compared to a hill-of-vision interpolation approach. This can facilitate early detection and monitoring of disease progression, ultimately guiding more personalized treatment strategies. In regions of EZ/IZ loss with hypertransmission, a hallmark of advanced dry AMD, these tailored grids could serve as a key functional endpoint for novel therapies, such as complement inhibition approaches targeting advanced dry AMD or assessing the conversion to late-stage AMD. For GA, targeted grids might be particularly useful for tracking new satellite lesions or monitoring the response to therapies. Longitudinal studies are needed to evaluate how changes in retinal sensitivity over time correlate with disease progression and treatment outcomes. Further research should also explore structure-function relationships to clarify how various structural changes impact retinal sensitivity.

OCT-based, targeted microperimetry grids provide a valuable tool for assessing the functional impact of focal lesions in iAMD. By improving the accuracy of retinal sensitivity measurements at areas of focal lesions, these customized grids enhance our understanding of disease progression and support the development of targeted therapeutic interventions.

## Supplementary Material

Supplement 1
