# Supplementary material for: Targeted Microperimetry Grids for Focal Lesions in Intermediate AMD: PINNACLE Study Report 7
Source: Invest Ophthalmol Vis Sci. 2025 Feb 4;66(2):6. doi: 10.1167/iovs.66.2.6 (PMC11801386; doi:10.1167/iovs.66.2.6)
Supplement: Supplement 1 [file iovs-66-2-6_s001.pdf]

Supplementary Material

Supplementary Table S1. *Linear mixed model outputs*

	Interpolation error (dB)		
Predictors	Estimates	95% CI	p
Sensitivity (dB)	-0.77	-0.83 – -0.71	**<0.001**
Focal lesion [Drusen collapse]	16.87	15.10 – 18.64	**<0.001**
Focal lesion [EZ/IZ loss + Hypertransmission]	13.60	12.52 – 14.69	**<0.001**
Focal lesion [Subretinal fluid]	9.17	5.77 – 12.57	**<0.001**
Distance to 24-points grid (°)	2.20	1.52 – 2.87	**<0.001**
Location [Peripheral]	-0.70	-1.20 – -0.19	**0.007**
Lesion size (mm^2)	-0.93	-2.60 – 0.74	0.273
Sensitivity (dB) × Focal lesion [EZ/IZ loss + Hypertransmission]	0.08	0.03 – 0.14	**0.003**
Sensitivity (dB) × Focal lesion [Subretinal fluid]	0.29	0.20 – 0.39	**<0.001**
Sensitivity (dB) × Distance to 24-points grid (°)	-0.08	-0.12 – -0.05	**<0.001**
Sensitivity (dB) × Location [Peripheral]	0.03	0.00 – 0.06	**0.024**

Focal lesion [EZ/IZ loss + Hypertransmission] × Lesion size (mm^2)	-19.41	-29.63 – -9.18	**<0.001**
Focal lesion [Subretinal fluid] × Lesion size (mm^2)	0.27	-2.60 – 3.15	0.852

Random Effects

σ^2	5.00
$\tau_{00\ \text{Focal lesion:Patient}}$	6.37
$\tau_{00\ \text{Patient}}$	8.22
ICC	0.74
$N_{\text{Focal lesion}}$	235
N_{Patient}	83
Observations	3025
Marginal R^2 / Conditional R^2	0.574 / 0.891

σ^2: Residual variance, $\tau_{00\ \text{Focal lesion:Patient}}$: Variance of lesion nested in patient, $\tau_{00\ \text{Patient}}$: Variance of patient

Supplementary Figure S1. *Alignment Validation Method*

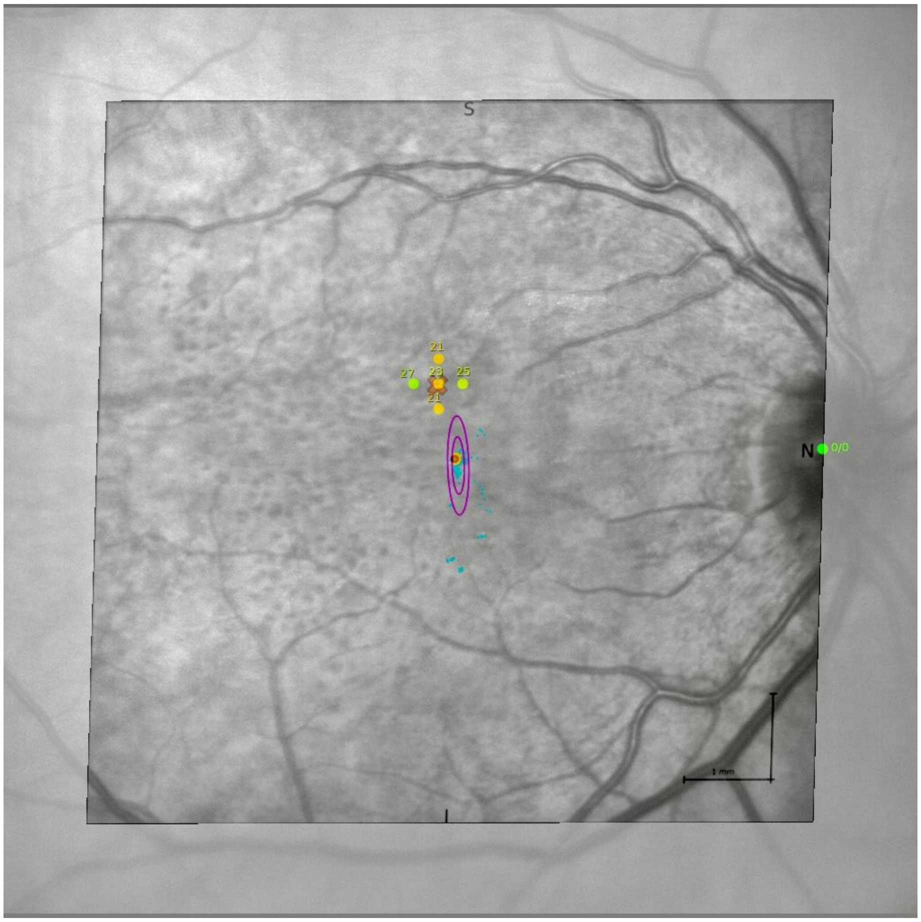

Overlay of infrared images acquired during OCT and microperimetry measurements, with the focal lesion highlighted by a red cross and the 5-point microperimetry grid depicted by points. The offset between the focal lesion's location and the central point of the 5-point microperimetry grid was measured and analyzed.

Supplementary Figure S2. *Alignment Validation Results*

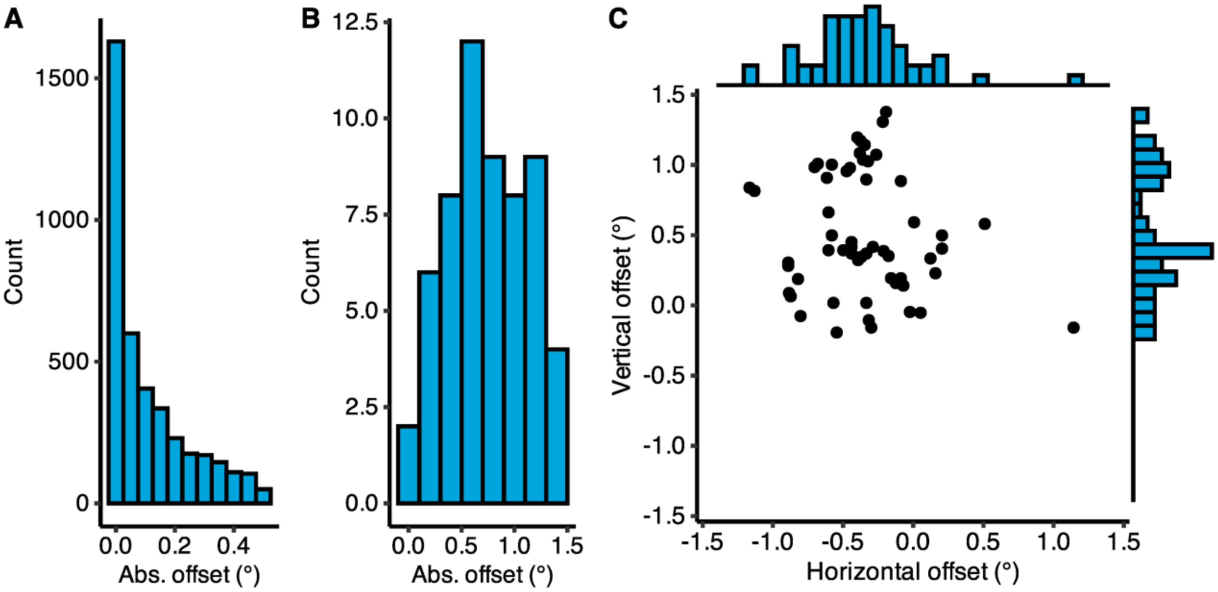

A. Histogram showing the offset between the focal lesion location and the central point in the 5-point microperimetry grid as indicated by the MAIA microperimetry device.

B. Histogram displaying the manually measured offset between the focal lesion location and the central point in the 5-point microperimetry grid for a random subset of data where the MAIA device indicated a 0° offset.

C. Scatter plot illustrating the horizontal and vertical offsets between the focal lesion location and the central point in the 5-point microperimetry grid, measured manually for a random subset of data where the MAIA device indicated a 0° offset.

Supplementary Figure S3. *Grid-Search Cross-Validation*

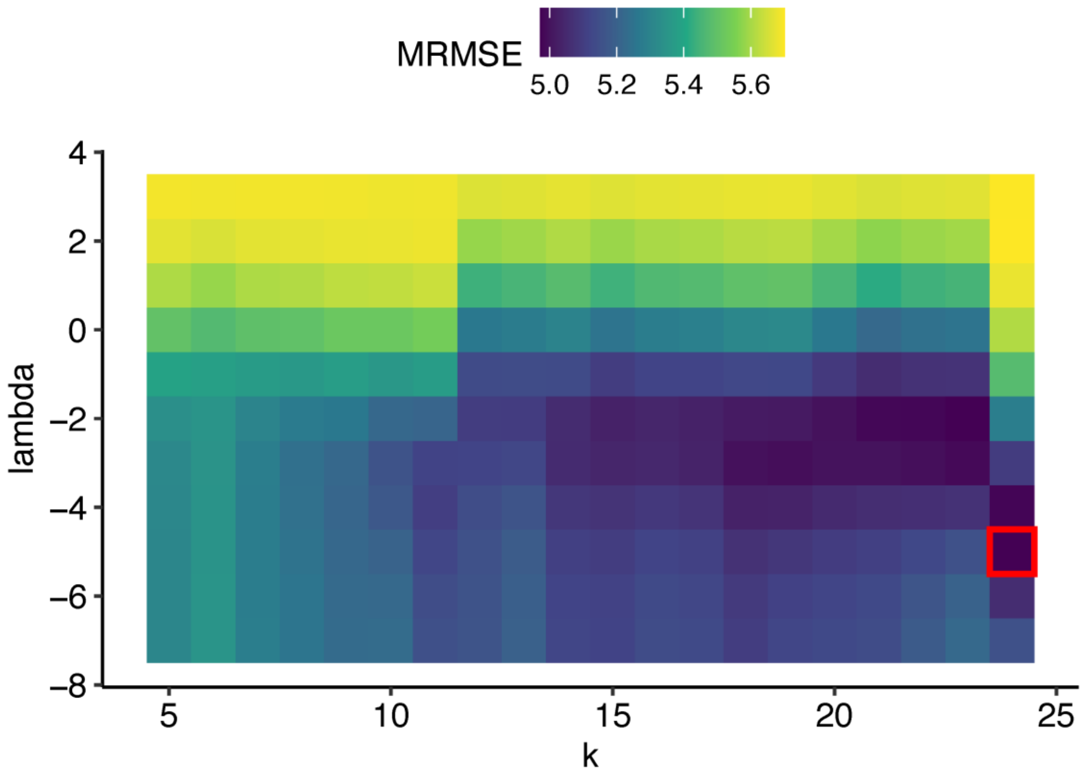

Tile plot illustrating the grid-search cross-validation results, showing the mean of the root mean squared errors (MRMSE) for different values of k and lambda. The red rectangular outline highlights the optimal choice of k and lambda that resulted in the lowest MRMSE.

Supplementary Figure S4. *Pearson Correlation*

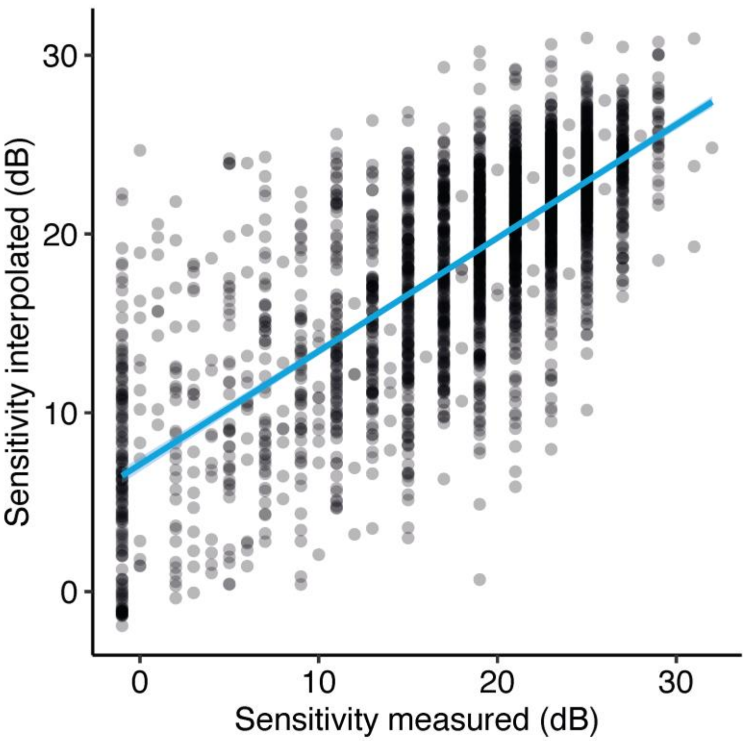

Scatter plot depicting the correlation between measured and predicted retinal sensitivity, with a blue regression line indicating the linear relationship.

Supplementary Equation S1. *Linear mixed model*

The following model was used for the analysis:

$$y = \beta_1 Sensitivity + \beta_2 Focal\ lesion + \beta_3 Distance + \beta_4 Location + \beta_5 Lesion\ size + \beta_6 (Sensitivity \times Focal\ lesion) + \beta_7 (Sensitivity \times Distance) + \beta_8 (Sensitivity \times Location) + \beta_9 (Focal\ lesion \times Lesion\ size) + u_{Patient} + u_{Lesion} + \epsilon$$

Where:

y is the error between measured and interpolation-derived focal sensitivity.

$\beta_1, \beta_2, \ldots\ \beta_9$ are the coefficients for the fixed effects.

$u_{Patient}$ represents the random effect for the patients.

u_{Lesion} represents the random effect for the nested factor ‘Focal lesion’ within ‘Patient’.

ϵ is the residual error term.

The coefficients are listed in the Supplementary Table 1.
